# Atom tunnelling in the reaction NH_3_^+^ + H_2_ → NH_4_^+^ + H and its astrochemical relevance†
†Electronic supplementary information (ESI) available: Rate constants, energies of the benchmark, coordinates of stationary points. See DOI: 10.1039/c6fd00096g



**DOI:** 10.1039/c6fd00096g

**Published:** 2016-05-31

**Authors:** Sonia Álvarez-Barcia, Marie-Sophie Russ, Jan Meisner, Johannes Kästner

**Affiliations:** a Institute for Theoretical Chemistry , University of Stuttgart , Pfaffenwaldring 55 , 70569 Stuttgart , Germany . Email: kaestner@theochem.uni-stuttgart.de ; Fax: +49-711-685-64442 ; Tel: +49-711-685-64473

## Abstract

The title reaction is involved in the formation of ammonia in the interstellar medium. We have calculated thermal rates including atom tunnelling using different rate theories. Canonical variational theory with microcanonically optimised multidimensional tunnelling was used for bimolecular rates, modelling the gas-phase reaction and also a surface-catalysed reaction of the Eley–Rideal type. Instanton theory provided unimolecular rates, which model the Langmuir–Hinshelwood type surface reaction. The potential energy was calculated on the CCSD(T)-F12 level of theory on the fly. We report thermal rates and H/D kinetic isotope effects. The latter have implications for observed H/D fractionation in molecular clouds. Tunnelling causes rate constants to be sufficient for the reaction to play a role in interstellar chemistry even at cryogenic temperature. We also discuss intricacies and limitations of the different tunnelling approximations to treat this reaction, including its pre-reactive minimum.

## 1 Introduction

The possible routes to NH_3_ formation in the interstellar medium are of significant interest in astrochemistry.^1^ One promising exothermic reaction is:

R1 NH_3_^+^ + H_2_ → NH_4_^+^ + H.

The resulting NH_4_^+^ species can generate ammonia and a hydrogen atom by recombining with an electron and subsequently dissociating a hydrogen atom.^1^(R1) is hindered by a barrier of 2.1 kcal mol^–1^ (8.8 kJ mol^–1^).^2^ The competitive dissociative recombination of NH_3_^+^ with an electron (formation of NH + H_2_) must be taken into account.^1^ This means that (R1) is most relevant in dark clouds where the concentration of free electrons is low.^3,4^

Early experiments reported a low rate constant of *k* < 5 × 10^–13^ cm^3^ s^–1^ for (R1) at room temperature.^2,5^ Smith and Adams measured the rate constant of (R1) down to approximately 80 K and found it to decrease with *T*.^6^ However, it is known that some exothermic ion–molecule reactions show an increase of reactivity by reducing the temperature.^7^ Experiments carried out at very low temperatures (20 to 11 K) by Luine and Dunn showed an increase in the reaction rate of (R1).^8^ They proposed a pre-reactive complex (NH_3_^+^ – H_2_) with an increased lifetime at lower temperature.^8^ The pressure-dependent results obtained by Böhringer supported this idea.^9^ In addition, the isotope effects detected by Smith and Adams,^6^ as well as Barlow and Dunn,^10^ also confirmed the presence of an entrance channel complex, from which tunnelling through the potential energy barrier could take place.^10,11^ Nevertheless, for a full understanding of reaction (R1) quantum chemical calculations are needed.

In 1991 Herbst *et al.* ^12^ mapped the potential energy surface (PES) for reaction (R1) at the MP4/6-311++G(3df,3dp)//MP2/6-31G(d,p) level.^13–17^ They found a weakly bound entrance channel complex (RS), as well as an exit channel complex (PS). Rate constants were calculated including a one-dimensional tunnelling approximation *via* an Eckart barrier. Their results qualitatively reproduced the variation of the rate coefficients with the temperature quite well, but they were quantitatively over 1.5 orders of magnitude lower than the experimental ones. Only by artificially reducing the potential energy barrier by 1 kcal mol^–1^ and increasing the curvature at the transition state, could they improve the agreement. Their predicted rate constants still remained lower than the experimental values; however, their calculations confirmed that tunnelling plays a significant role in reaction (R1) at low temperatures.

The main goal of this study is to calculate tunnelling rates with more accurate methods than the ones found in the literature.^12,18^ Firstly, a careful study of the PES comparing high-level methods has been done. Secondly, bimolecular rate constants were calculated with transition state theory including tunnelling through several approaches,^19^ and unimolecular rate constants were additionally computed with the instanton method.^20–37^ Finally, calculations regarding the kinetic isotope effect were carried out.

## 2 Computational details

### 2.1 Optimisation procedure

All the geometry optimisations were performed with DL-FIND^38^ in ChemShell.^39,40^ For the electronic structure computations (energy and gradient) we have employed Molpro 2012.^41^ Starting points for the geometries were obtained from Herbst *et al.* ^12^

We carried out several single point calculations using the geometries obtained at the MCSCF/cc-pVTZ level.^42,43^ We used the MRCISD(Q)-F12/cc-pVQZ-F12//MCSCF/cc-pVTZ level^42–45^ as a reference and compared the relative energies of the RS, TS and PS of reaction (R1) (see the ESI).† Table S1 and Fig. S1† show that the relative energies at the UCCSD(T)-F12a/cc-pVDZ-F12//MCSCF/cc-pVTZ level^42,43,45–47^ agree well with the reference results. In addition, the analysis of the *T*_1_-diagnostic values for the coupled cluster calculations allows us to conclude that a single-reference wave function can be used to describe this system, since the *T*_1_ values were always lower than 0.02.^48,49^ For all further calculations we have used the UCCSD(T)-F12a/cc-pVDZ-F12 level,^45–47^ which represents a reasonable compromise between accuracy and computational efficiency.

The default thresholds of the programs were used in general. However, for the optimisation of the TS, we employed stricter convergence thresholds in order to reproduce the *C*_3v_ symmetry for the TS and the correct eigenvalue spectrum of the Hessian. We changed the maximum gradient of the geometry optimisation in DL-FIND to 5 × 10^–6^ atomic units. Additionally, the threshold of the SCF and UCCSD energy convergence was set to 10^–10^ Hartree for the optimisation of the stationary points.

### 2.2 Variational transition state theory calculations

We employed TST by using the Polyrate code version 2010.^50^ We used conventional TST as well as the canonical variational TST (CVT) (the position of the TS is located variationally for every temperature). Furthermore, the transmission factors were tested with several tunnelling methods (in combination with the TST types): zero-curvature tunnelling method (ZCT), small-curvature tunnelling method (SCT), large-curvature tunnelling method (LCT), canonically-optimised multidimensional tunnelling method (COMT) and the microcanonically-optimised multidimensional tunnelling method (μOMT).^19,51^ In the COMT method, the transmission factor corresponds to the largest thermally averaged value between the SCT and LCT for a given *T*. In the μOMT method, the transmission factor is computed from LCT and SCT results by taking the larger of the two for each energy. For (R1), SCT always results in larger transmission factors than LCT.

The minimum energy path (MEP) was calculated at the UCCSD(T)-F12/cc-pVDZ-F12 level. The Page–McIver algorithm^52^ (implemented in Polyrate) was used to follow the reaction path, with the Hessian recalculated every 9 steps. To be able to use energy and gradients in Polyrate directly from Molpro, we interfaced Polyrate to ChemShell. We improved the accuracy by using the ISPE approach (interpolated single-point energies):^50,53^ the energy of the stationary points and some points along the MEP were recalculated with a larger basis set (UCCSD(T)-F12/cc-pVTZ-F12, see Fig. S1†). Note that the calculations have been done considering both the wells in the reactant region (RS) and the product side (PS).

Therefore, for the rate constant calculations including tunnelling by multidimensional transmission (MT) factors, we considered a pre-reactive complex called RS (see details in Sections 3.1 and 3.2).

An important detail in the rate calculations is to include the symmetry factor (*σ*) which is related to the symmetry index of the reactants and saddle point:^54^
1
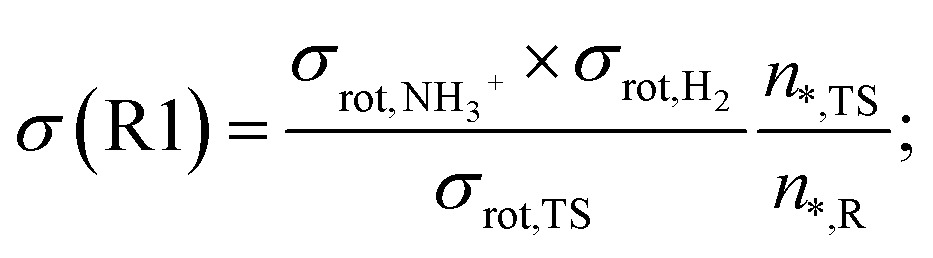

*σ*_rot_ represents the rotational symmetry number and n_*_ the number of optical isomers of the molecule. For (R1): *σ*_rot_(NH_3_^+^, *D*_3h_) = 6; *σ*_rot_(H_2_, *D*_∞h_) = 2 and *σ*_rot_(TS, *C*_3v_) = 3. Consequently, *σ*(R1) = 4. For the unimolecular reaction from the pre-reactive complex RS, *σ*(RS, *C*_s_), the overall symmetry factor is *σ* = 1/3.

### 2.3 Instanton calculations

We also used the instanton method^20–37^ based on Feynman path integral theory using the semiclassical approximation to obtain the rate constants. For a given temperature, it provides the most probable tunnelling path, the instanton. Instanton theory is applicable whenever the temperature is low enough for the instanton to spread out. At higher temperatures, the instanton collapses to a point which renders the theory inapplicable. For many barrier shapes^55^ this collapse happens at the crossover temperature *T*_c_,^56^
2
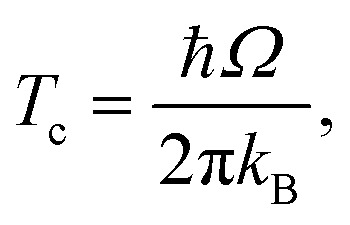
with *Ω* being the barrier frequency (the absolute value of the imaginary frequency corresponding to the transition mode) and *k*_B_ corresponding to Boltzmann's constant.

Instanton paths were optimised *via* a quasi Newton–Raphson method as described previously.^34,35^ Energies and gradients were provided by Molpro, but instanton optimisations and Hessian calculations *via* finite differences were done in DL-FIND. The instanton path was discretised using 154 images.

## 3 Results

### 3.1 PES calculations

**Fig. 1:**
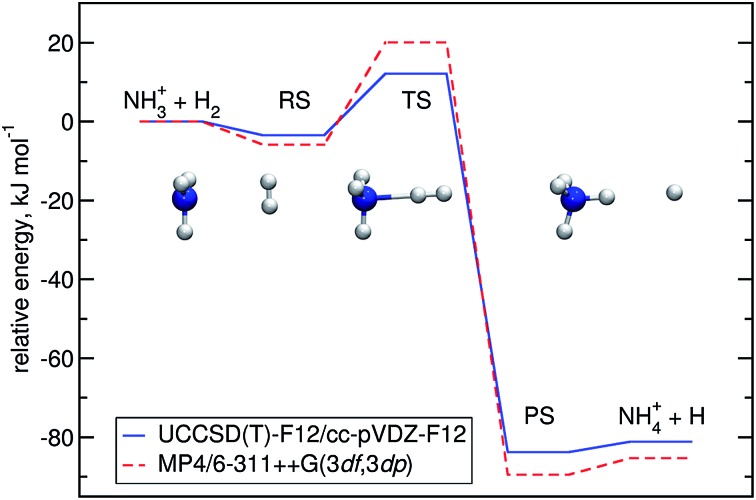
Energy profile of reaction (R1) computed here (blue) compared to a previous one^12^ (red). The relative energies (kJ mol^–1^) including ΔZPEs of the RS, TS, PS and products were calculated with respect to the reactants.

Optimisation of the geometries for the stationary points in reaction (R1) was performed at the UCCSD(T)-F12/cc-pVDZ-F12 level. We found a pre-reactive complex (RS) with the dihydrogen molecule almost parallel to the plane of the NH_3_ fragment with N–H distances of 2.619 Å and 2.605 Å, see Fig. 1. The RS is 8.57 kJ mol^–1^ below the energy of the separated reactants (3.25 kJ mol^–1^ when the ZPE is included). In the *C*_3v_-symmetric transition state structure, H_2_ attacks the nitrogen atom head-on, with an N–H distance of 1.647 Å and a H–H distance of 0.786 Å. Compared to the separated reactants, the barrier is 6.03 kJ mol^–1^ (12.13 kJ mol^–1^ when the ZPE is included). Cartesian coordinates of all stationary points are available in the ESI.† A comparison to the previous potential energy surface on the MP4/6-311++G(3df,3dp)//MP2/6-31G(d,p) level^12^ is given in Fig. 1. It is clear that there is a significant pre-reactive minimum, which can be expected to be populated at low temperature and high-enough pressure to allow for thermal equilibration within the lifetime of the complex.

### 3.2 Bimolecular rate constants

To obtain bimolecular reaction rates, we have employed TST (also in its canonical version) in combination with more or less sophisticated tunnelling methods. Note that the variational determination of the transition state (model CVT *vs.* TST) has a negligible impact on the reaction rates. As has been said, the small curvature results, SCT, always give higher transmission factors than their LCT counterparts, so the CVT/COMT (and μOMT) results are equal to the CVT/SCT values; furthermore, the μOMT and COMT results are coincident. A full list of results is given in Table S2 of the ESI.†

In addition, we have employed the ISPE correction to improve our results, see Table S3 of the ESI.† The energies of the stationary points and some points along the path have been recalculated with the larger cc-pVTZ-F12 basis set. This lowers the barrier by 0.9 kJ mol^–1^. ISPE is not available for CVT/LCT, but we assume that the rates remain smaller than those of the CVT/SCT approach.

**Fig. 2:**
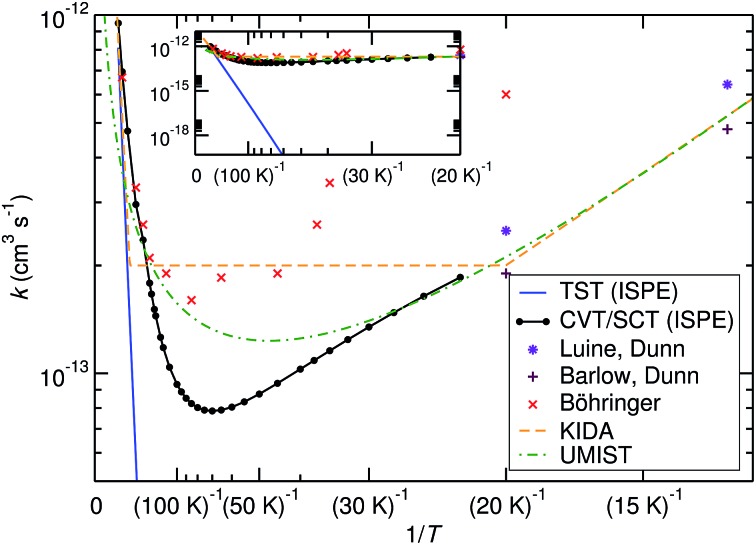
Comparison of calculated bimolecular rate constants to experimental values and ref. 9–11. The inset shows the same graphs at different scaling and demonstrates the effect of tunnelling.

The comparison of the TST and CVT/SCT rate constant values (including the ISPE correction) (see Fig. 2) shows that the reaction proceeds mostly classically above approximately 200 K. We can observe a monotonic increase in rate with temperature at *T* > 200 K. Therefore, in that *T*-range, the CVT/SCT values are practically coincident with the TST values. However, tunnelling dominates below the crossover temperature of *T*_c_ = 148.5 K. The rate constant is minimal at 70 K and slightly increases at even lower temperature. In full thermal equilibrium, this could be caused by the population of a bound pre-reactive complex (RS), which increases the attempt frequency for the transition.^57^ At low pressure, however, one would expect the RS to decay to the products or back to the reactants before thermal equilibration. Note that the Polyrate code^50^ does not compute the capture rates (formation of the RS complex from NH_3_^+^ + H_2_). However, the presence of the RS pre-reactive complex is taken into account (and it has a huge impact) during the MEP calculation. This MEP is required for the CVT rate constant and MT tunnelling factor calculations. A good description of the path is necessary because it has a large impact in the selection of the turning points during the tunnelling calculation.

#### 3.2.1 Comparison to experiments

The rate constant of the title reaction was measured several times in different temperature ranges.^2,5,6,8–11^ We have summarised these results in Fig. 2 and compared them to our results and the values used in the astrochemical databases UMIST and KIDA.^58,59^

Before comparing to theory, it makes sense to compare the experimental results among each other. At 20 K, the rate constant obtained by Böhringer^9^ is 2.4 times larger than the one by Luine and Dunn,^8^ and 3.2 times larger than the one by Barlow and Dunn.^10^ Our results, extrapolated to 20 K, can be expected to be close to those by Luine and Dunn, as well as those by Barlow and Dunn, see Fig. 2. Consequently, one may speculate that the higher-temperature values by Böhringer somewhat overestimate the rate constant as well. Our high-*T* rate constants agree quite well with the experimental ones. There is also agreement that the rate constant has a minimum in the temperature range between about 100 K and 30 K. Below that, the rate constant increases again, which is found by both theory and experiment.

The rate expressions used in the kinetic databases KIDA and UMIST agree remarkably well with our results at low temperature. Also at high temperature, the expression used in KIDA agrees well with our data, while UMIST somewhat underestimates the rate constant. At intermediate temperatures, where the rate constant is minimal, KIDA, UMIST and our results are always within a factor of 3.

#### 3.2.2 Instanton calculations: intricacies and limitations

**Fig. 3:**
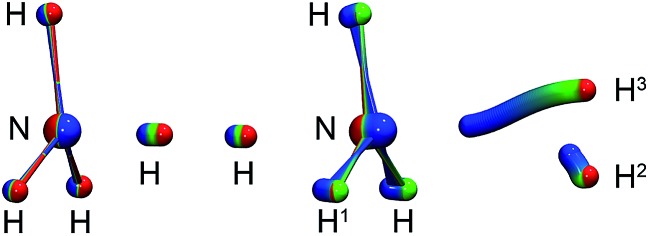
Left: instanton at 145 K (*C*_3v_). Right: instanton at 50 K (*C*_s_). Images are coloured from the RS-end (red) to the PS-end (blue).

The crossover temperature (*T*_c_) for this reaction is 148.5 K. Instantons were calculated in the range of 145 to 50 K. The PES for this reaction leads to several difficulties for semiclassical instanton theory. The first is the difference in symmetry between the TS and the RS. The instanton at 145 K shows a relatively small delocalisation and retains the *C*_3v_ symmetry of the TS, see Fig. 3. However, by lowering *T* to just 140 K, the delocalisation increases and the *C*_3v_-symmetric instanton becomes unstable. A symmetry breaking takes place; first-order saddle point paths in the Euclidean action at lower temperature have the *C*_s_ symmetry of the RS. Similar temperature-dependent symmetry breakings have also been observed in other cases.^60^ In the temperature range of the instability between the instantons of different symmetry, the vibrational frequency of the instanton associated with the instability becomes small, leading to unrealistically high predicted rate constants. Combined with the previous finding^61^ that instanton theory often overestimates the rate constant close to *T*_c_, instanton rates are probably only reliable well below *T*_c_.

**Fig. 4:**
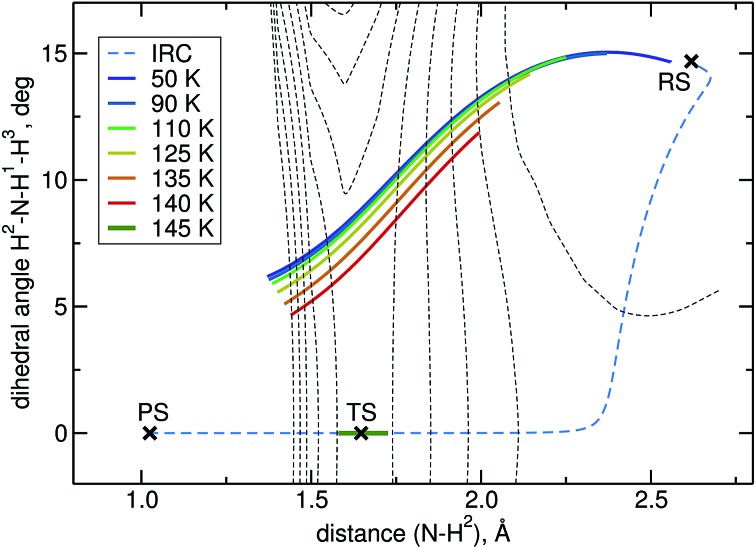
Symmetry breaking in instanton calculations: the plot shows instantons, the IRC and the stationary points projected onto a dihedral angle and the N–H bond length. *C*_3v_ symmetry corresponds to a dihedral angle of 0. Equipotential lines at –3, –1, 1, … kJ mol^–1^ are shown as thin dashed lines.

The intrinsic reaction coordinate (IRC) is *C*_3v_-symmetric at the TS and quite some way down into the reactant well, see Fig. 4. There, a dihedral angle which shows the distortion from *C*_3v_ symmetry is plotted against the distance of the attacking hydrogen atom to the nitrogen atom for the IRC and several instanton paths. The instanton paths for *T* ≤ 140 K differ substantially from the IRC. This phenomenon is known as corner cutting by the tunnelling motion.^62^ It can also be seen in Fig. 4 that the lower the temperature gets, the longer the instanton paths become. The fact that they end in similar areas of configuration space on the product side is due to the fact that both ends of the instanton path have the same energy. So they end when the energy of the PS side drops below the energy of the RS. Here, it should be noted that only half of each instanton path is visible in Fig. 4: the paths are closed on themselves and follow the line shown in Fig. 4 backward and forwards.

A different problem in instanton calculations concerns the evaluation of bimolecular rates. Since the barrier is rather low (almost submerged) compared to the RS, all instantons for *T* < 145 K have a tunnelling energy (the energy of the turning points of the instanton) below the energy of the separated reactants. Therefore, bimolecular rate constants are not available from instanton theory. It would be possible to calculate temperature-dependent bimolecular rates by calculating energy-dependent transmission coefficients and taking the Boltzmann average of these based on the energy of the separated reactants. This was outside the scope of this paper, though. Instead, we calculated unimolecular rate constants.

### 3.3 Unimolecular rate constants

The title reaction is expected to be relevant on the surface of interstellar dust grains, where unimolecular rate constants are relevant. Different types of surface reactions can be distinguished: if one reaction partner is adsorbed on the surface and the other one comes in from the gas phase, one speaks of Eley–Rideal-type reactions. Those are described by bimolecular rate constants. If both partners are adsorbed on the surface and move diffusively until they meet, a Langmuir–Hinshelwood mechanism takes place. The corresponding rate constants can be approximated by unimolecular gas-phase rate constants from the RS minimum. Adsorption of one or both reactants on a dust grain can, of course, change the PES and especially the barrier height. For a reasonably inert surface this effect might be neglected in a first approximation. The other effect of adsorption is the greatly facilitated thermal equilibration. Excess heat produced by formation of a pre-reactive complex or by the reaction itself is rapidly dissipated into the bulk, which justifies the use of thermal rate constants.

**Fig. 5:**
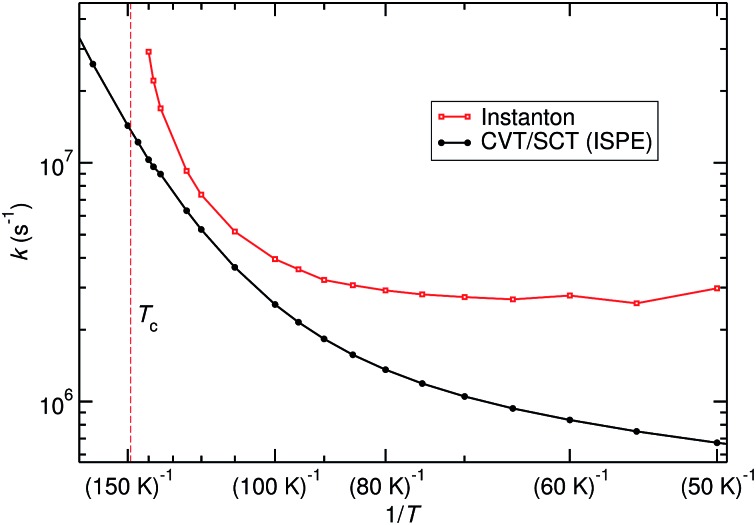
Unimolecular rate constants calculated with instanton theory compared to CVT/SCT.

Unimolecular rate constants were calculated with instanton theory and CVT/SCT theory. Results are shown in Fig. 5 and Table S4 of the ESI.† At high temperatures, close to *T*_c_, instanton theory is known to overestimate the rate constant.^61^ This is also seen here. Between 120 K and 80 K there is reasonable agreement between the methods. At low temperature, instanton theory predicts a temperature-independent unimolecular rate constant, while the one predicted by CVT/SCT continues to decrease. Below 80 K, one can assume instanton rate constants to be more trustworthy than the SCT data because instanton theory uses an optimised tunnelling path. As shown in Fig. 4, the tunnelling path differs significantly from the IRC at low temperature. It should be noted that the instanton optimisations for *T* < 60 K are not perfectly converged, which causes the slight wiggles in Fig. 5.

### 3.4 Kinetic isotope effects

We have also studied the effect of replacing H_2_ by HD and D_2_ in (R1), the kinetic isotope effect (KIE). This influences the deuterium fractionation of molecules observed in the interstellar medium.^4,63^ The KIE is defined as the ratio between the rate constant of a reaction with a lighter isotope and the rate constant of the reaction with a heavier isotope. The following reactions were investigated:

R2 NH_3_^+^ + HD → NH_4_^+^ + D,

R3 NH_3_^+^ + DH → NH_3_D^+^ + H,

R4 NH_3_^+^ + D_2_ → NH_3_D^+^ + D.

Note that (R4) has been studied experimentally by Adams and Smith, as well as Barlow and Dunn.^10,11^(R2) and (R3) both represent the reaction of NH_3_^+^ with HD, but the products are different. From the resulting rate constant, one can calculate a branching ratio.

**Fig. 6:**
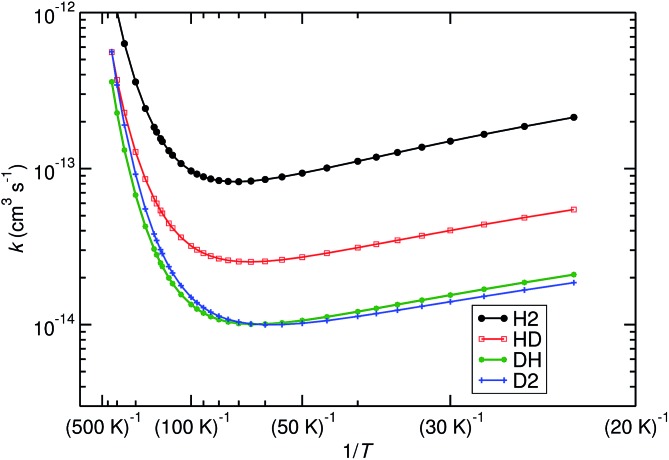
Effect of deuteration on the reaction rates, calculated with TST/SCT, ISPE correction at the UCCSD(T)-F12/cc-pVTZ-F12 level. Reactions (R1) (noted as H_2_, black), (R2) (noted as HD, red), (R3) (noted as DH, green) and (R4) (noted as D_2_, blue) are plotted.

We have computed the rates of the deuterated systems with Polyrate, which includes a scaling procedure to recalculate the rates from the non-deuterated reactions.^64,65^ TST in combination with the SCT method (TST/SCT), including the ISPE correction, was used. The approach is almost equivalent to the one employed in Fig. 2, with only CVT being replaced by TST. For the non-deuterated case, these two methods resulted in almost identical rate constants, so we assume that they are very similar for the deuterated cases as well. The most important results are shown in Fig. 6. Furthermore, some KIE values are listed in Table 1.

**Table 1:** KIEs values for reactions (R2)–(R4) and branching ratio (BR) for (R2)*vs.*(R3)

*T*, K	KIE (HD)	KIE (DH)	BR (%)	KIE (D_2_)	KIE (D_2_) ref. 11
25	3.8	10.0	72	11.2	—
35	3.7	9.4	72	10.3	—
45	3.5	9.0	72	9.5	—
55	3.4	8.6	72	8.8	—
65	3.3	8.3	72	8.2	—
75	3.2	7.9	71	7.7	—
85	3.1	7.6	71	7.1	11.3
100	3.0	7.2	71	6.5	—
110	3.0	6.9	70	6.1	—
125	2.9	6.5	69	5.5	—
145	2.9	6.1	68	5.0	—
170	2.8	5.7	67	4.4	—
200	2.8	5.3	65	3.9	3.3
300	2.7	4.5	63	2.9	1.1

## 4 Conclusions

We have calculated bimolecular and unimolecular rate constants for the title reaction with different rate theories. Our results agree to a reasonable degree with available experimental data. They also confirm the fitted rate expressions currently used in astrochemical models. It is clear that at low temperatures (<70 K), the bimolecular rate constant increases with decreasing temperature. This unusual behaviour is caused by the quantum mechanical tunnel effect. Without tunnelling (see the TST graph in Fig. 2), the rate constant decreases strongly at lower temperature.

It is clear that atom tunnelling plays a key role in the title reaction. The shape of the potential energy surface, including an almost submerged barrier and a tunnelling path which differs significantly from the minimum-energy path, lead to challenges in the theoretical description. For the title reaction, instanton theory is only applicable well below the crossover temperature. In that region, however, one can expect it to provide more accurate rate constants than CVT/SCT. The latter assumes a tunnelling path close to the minimum-energy path by construction. It accounts for some corner cutting, but can not adapt to qualitatively different tunnelling paths. Nevertheless, the agreement with experiment is quite satisfactory.
