# Supplementary material for: Atom tunnelling in the reaction NH3 + + H2 → NH4 + + H and its astrochemical relevance
Source: Faraday Discuss. 2016 May 31;195(0):69–80. doi: 10.1039/c6fd00096g (PMC5317219; doi:10.1039/c6fd00096g)
Supplement: Supplementary file 1 [file FD-195-C6FD00096G-s001.pdf]

Electronic Supplementary Material (ESI) for Faraday Discussions.
This journal is © The Royal Society of Chemistry 2016

SUPPORTING INFORMATION

Atom tunnelling in the reaction $NH_3^+ + H_2 \longrightarrow NH_4^+ + H$ and its astrochemical relevance

Sonia Álvarez-Barcia, Marie-Sophie Russ, Jan Meisner and Johannes Kästner

Table S1 Relative energies in kJ/mol of the RS, TS and PS of reaction R1 and the root mean square (RMSD) of these energies with respect to the energies obtained at the MRCISD-F12/cc-pVQZ-F12//MCSCF/cc-pVTZ level (including Davidson correction (DC)). Note that the single point calculations were done using the geometries obtained at the MCSCF/cc-pVTZ level.

Method	Basis set	Relative energies			RMSD
		RS	TS	PS	
UCCSD(T)-F12a	cc-pVDZ	-6.53	7.11	-107.16	1.83
	cc-pVTZ	-7.40	5.40	-106.13	0.63
	cc-pVQZ	-7.69	4.90	-106.48	0.74
	cc-pVDZ-F12a	-7.47	5.90	-104.45	0.74
	cc-pVTZ-F12a	-7.80	4.94	-105.52	0.18
	cc-pVQZ-F12a	-7.85	4.78	-106.24	0.61
	aug-cc-pVDZ	-7.96	5.01	-106.13	0.56
	aug-cc-pVTZ	-7.93	4.74	-106.17	0.58
	aug-cc-pVQZ	-7.90	4.67	-106.58	0.82
MRCISD without DC	cc-pVTZ	-1.71	13.21	-99.66	6.72
	cc-pVQZ	-2.34	12.09	-102.09	5.48
MRCISD-F12 without DC	cc-pVDZ-F12	-2.25	11.61	-101.81	5.36
	cc-pVTZ-F12	-2.61	11.47	-102.75	5.00
	cc-pVQZ-F12	-2.67	11.52	-103.31	4.92
MRCISD with DC	cc-pVTZ	-6.18	7.35	-101.12	2.88
	cc-pVQZ	-7.17	5.68	-103.84	0.96
MRCISD-F12 with DC	cc-pVDZ-F12	-7.11	5.14	-103.68	0.95
	cc-pVTZ-F12	-7.59	4.84	-104.63	0.34
	cc-pVQZ-F12	-7.67	4.88	-105.20	0.00
MCSCF	cc-pVTZ	-4.07	23.93	-124.90	15.68
	cc-pVQZ	-4.72	24.23	-125.19	15.95
	cc-pV5Z	-4.89	24.36	-125.18	16.09
	aug-cc-pVQZ	-4.97	24.25	-125.28	16.15
	aug-cc-pV5Z	-4.94	24.36	-125.24	16.18
	cc-pVDZ-F12	-4.44	23.96	-124.27	16.18
	cc-pVTZ-F12	-4.85	24.28	-125.02	16.19
	cc-pVQZ-F12	-4.93	24.37	-125.16	16.21
CASPT2	cc-pVTZ	-6.53	6.96	-104.02	1.53
	cc-pVQZ	-7.51	5.54	-105.57	0.45
	cc-pV5Z	-7.84	5.16	-106.02	0.51
	aug-cc-pVTZ	-8.09	5.16	-105.29	0.30
	aug-cc-pVQZ	-8.08	4.89	-105.97	0.50
	aug-cc-pV5Z	-8.02	4.89	-106.18	0.60
DF-CASPT2	cc-pVDZ	-4.27	10.50	-105.74	3.80
	cc-pVTZ	-6.54	7.00	-103.89	1.58
	cc-pVQZ	-7.51	5.56	-105.52	0.44
	cc-pV5Z	-7.85	5.13	-106.06	0.52

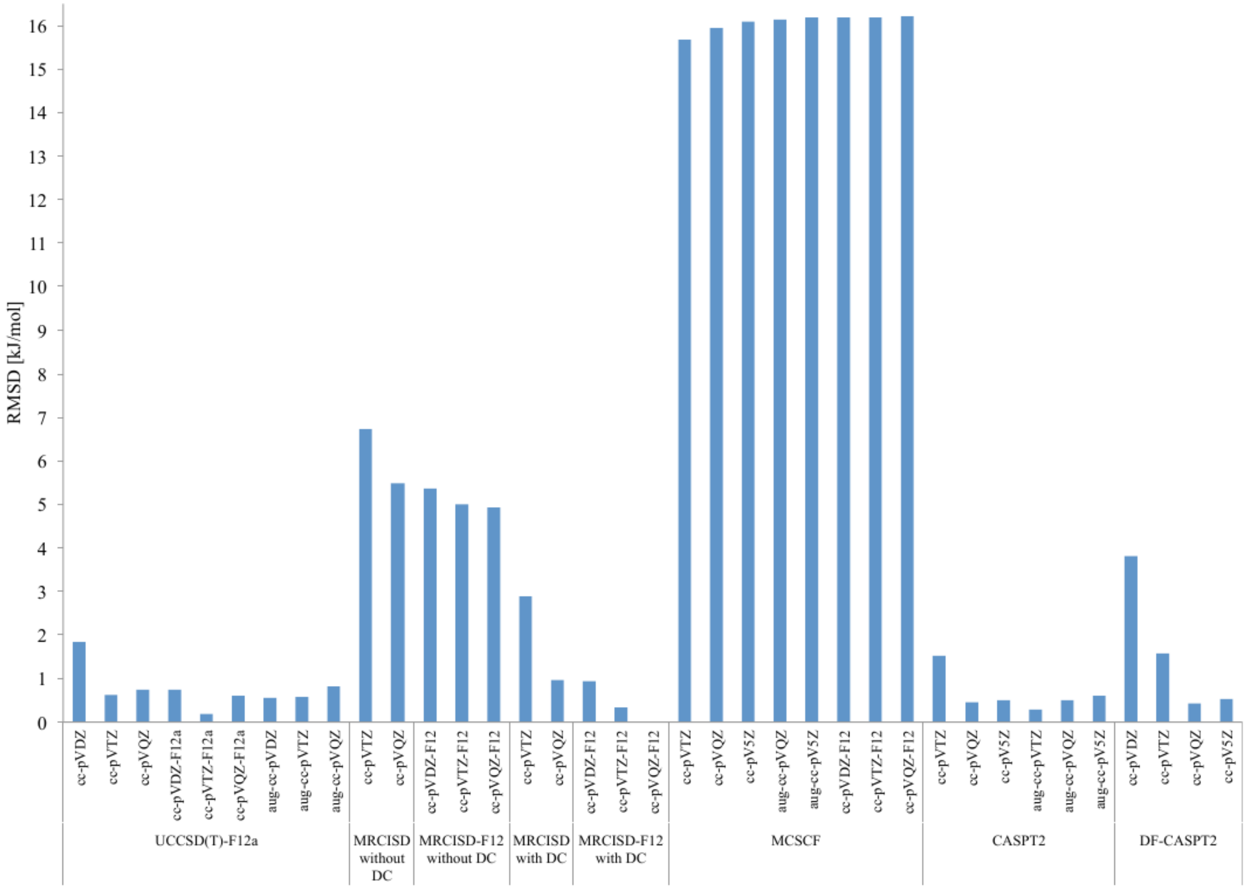

Figure S1 RMSD of the energies listed in table S1 with respect to the MRCISD-F12/cc-pVQZ-F12//MCSCF/cc-pVTZ energy.

Cartesian coordinates of all the stationary points.

NH_3^+

N -3.316971 1.830514 0.002968
H -2.354318 2.170552 -0.014804
H -3.797924 1.705481 0.895045
H -3.798735 1.615534 -0.871278

H_2

H -2.422087 3.890101 0.000000
H -1.683813 3.964039 0.000000

NH_4^+

N -2.036678 1.055785 0.006008
H -1.015432 1.063308 -0.003987
H -2.365471 0.165612 0.383672
H -2.388627 1.172473 -0.945600
H -2.377200 1.820576 0.591175

RS

N -2.1857779 2.5632825 -0.3380093
H -1.2361175 2.2579245 -0.1254340
H -2.6718496 2.1869022 -1.1519603
H -2.6524804 3.2396802 0.2661371
H -2.8634046 0.8489791 1.5225391
H -3.1990561 0.5297622 0.9361751

TS

N -2.208348 2.508710 -0.284435
H -1.214508 2.321618 -0.162378
H -2.651627 2.216800 -1.153815
H -2.620863 3.284045 0.231530
H -2.775747 1.290274 0.666826
H -3.046733 0.708359 1.120601

PS

N -2.39088 2.22590 0.00000
H -1.37088 2.22590 0.00000
H -2.73088 2.15010 -0.95867
H -2.73089 3.09404 0.41368
H -2.73052 1.45329 0.57539
H -3.05426 0.83558 1.05783

Table S2 TST/CVT rates (Log(*k*)) for reaction R1 (computed with several tunneling models) employed a CCSD(T)-F12/ccpVTZ-F12 reaction coordinate.

T(K)	1000/T (1/K)	TST	CVT	TST/SCT	TST/LCT	TST/COMT	CVT/SCT	CVT/LCT	CVT/COMT
22.50	44.44	-37.14	-37.19	-12.97	-13.49	-12.97	-12.99	-13.51	-12.99
25.00	40.00	-34.40	-34.44	-13.02	-13.54	-13.02	-13.04	-13.56	-13.04
27.50	36.36	-32.16	-32.20	-13.07	-13.58	-13.07	-13.09	-13.60	-13.09
30.00	33.33	-30.30	-30.34	-13.11	-13.62	-13.11	-13.13	-13.63	-13.13
32.50	30.77	-28.73	-28.77	-13.15	-13.65	-13.15	-13.16	-13.67	-13.16
35.00	28.57	-27.39	-27.43	-13.18	-13.68	-13.18	-13.19	-13.69	-13.19
37.50	26.67	-26.23	-26.27	-13.21	-13.71	-13.21	-13.22	-13.72	-13.22
40.00	25.00	-25.22	-25.25	-13.23	-13.73	-13.23	-13.24	-13.74	-13.24
45.00	22.22	-23.54	-23.57	-13.27	-13.76	-13.27	-13.28	-13.77	-13.28
50.00	20.00	-22.21	-22.23	-13.30	-13.79	-13.30	-13.31	-13.80	-13.31
55.00	18.18	-21.12	-21.14	-13.33	-13.81	-13.33	-13.34	-13.82	-13.34
60.00	16.67	-20.22	-20.24	-13.34	-13.81	-13.34	-13.35	-13.82	-13.35
65.00	15.38	-19.46	-19.48	-13.35	-13.82	-13.35	-13.36	-13.82	-13.36
70.00	14.29	-18.81	-18.83	-13.35	-13.81	-13.35	-13.36	-13.82	-13.36
75.00	13.33	-18.25	-18.27	-13.35	-13.80	-13.35	-13.36	-13.81	-13.36
80.00	12.50	-17.76	-17.78	-13.34	-13.78	-13.34	-13.35	-13.79	-13.35
85.00	11.76	-17.34	-17.35	-13.33	-13.76	-13.33	-13.34	-13.77	-13.34
90.00	11.11	-16.96	-16.97	-13.31	-13.74	-13.31	-13.32	-13.74	-13.32
95.00	10.53	-16.62	-16.63	-13.29	-13.71	-13.29	-13.30	-13.71	-13.30
100.00	10.00	-16.32	-16.33	-13.27	-13.67	-13.27	-13.28	-13.68	-13.28
110.00	9.09	-15.80	-15.81	-13.22	-13.60	-13.22	-13.23	-13.61	-13.23
120.00	8.33	-15.36	-15.37	-13.16	-13.51	-13.16	-13.16	-13.52	-13.16
125.00	8.00	-15.17	-15.18	-13.13	-13.47	-13.13	-13.13	-13.47	-13.13
135.00	7.41	-14.84	-14.85	-13.06	-13.38	-13.06	-13.07	-13.39	-13.07
138.00	7.25	-14.75	-14.76	-13.04	-13.35	-13.04	-13.05	-13.36	-13.05
145.00	6.90	-14.55	-14.56	-13.00	-13.29	-13.00	-13.00	-13.30	-13.00
150.00	6.67	-14.42	-14.43	-12.96	-13.25	-12.96	-12.98	-13.26	-12.98
170.00	5.88	-13.98	-14.00	-12.83	-13.07	-12.83	-12.89	-13.13	-12.89
200.00	5.00	-13.48	-13.52	-12.64	-12.84	-12.64	-12.72	-12.92	-12.72
250.00	4.00	-12.91	-12.99	-12.36	-12.50	-12.36	-12.49	-12.63	-12.49
300.00	3.33	-12.52	-12.63	-12.14	-12.24	-12.14	-12.30	-12.40	-12.30
350.00	2.86	-12.23	-12.37	-11.95	-12.03	-11.95	-12.15	-12.23	-12.15

Table S3 TST/CVT rates ((Log(k)) for reaction R1 (computed with several tunneling models) employed a CCSD(T)-F12/ccpVDZ-F12 reaction coordinate corrected with the interpolated single-point energies (ISPE) scheme (single point energy calculations along the path at the CCSD(T)-F12/ccpVTZ-F12 level).

T(K)	1000/T (1/K)	TST	CVT	TST/SCT	CVT/SCT
22.50	44.44	-35.04	-35.10	-12.67	-12.73
25.00	40.00	-32.51	-32.57	-12.73	-12.79
27.50	36.36	-30.44	-30.49	-12.78	-12.83
30.00	33.33	-28.73	-28.77	-12.82	-12.87
32.50	30.77	-27.28	-27.32	-12.86	-12.91
35.00	28.57	-26.04	-26.08	-12.90	-12.94
37.50	26.67	-24.97	-25.01	-12.93	-12.96
40.00	25.00	-24.04	-24.08	-12.95	-12.99
45.00	22.22	-22.49	-22.53	-13.00	-13.03
50.00	20.00	-21.26	-21.29	-13.03	-13.06
55.00	18.18	-20.26	-20.29	-13.05	-13.08
60.00	16.67	-19.43	-19.45	-13.07	-13.09
65.00	15.38	-18.73	-18.75	-13.08	-13.10
70.00	14.29	-18.13	-18.16	-13.08	-13.11
75.00	13.33	-17.62	-17.64	-13.08	-13.10
80.00	12.50	-17.17	-17.19	-13.08	-13.10
85.00	11.76	-16.78	-16.80	-13.07	-13.08
90.00	11.11	-16.43	-16.45	-13.05	-13.07
95.00	10.53	-16.12	-16.14	-13.04	-13.05
100.00	10.00	-15.84	-15.86	-13.02	-13.03
110.00	9.09	-15.36	-15.38	-12.97	-12.98
120.00	8.33	-14.97	-14.98	-12.91	-12.93
125.00	8.00	-14.80	-14.81	-12.89	-12.90
135.00	7.41	-14.49	-14.50	-12.83	-12.84
138.00	7.25	-14.40	-14.42	-12.81	-12.82
145.00	6.90	-14.22	-14.23	-12.77	-12.78
150.00	6.67	-14.10	-14.12	-12.74	-12.75
170.00	5.88	-13.70	-13.71	-12.61	-12.63
200.00	5.00	-13.24	-13.27	-12.44	-12.53
250.00	4.00	-12.72	-12.78	-12.20	-12.32
300.00	3.33	-12.36	-12.45	-11.99	-12.16
350.00	2.86	-12.09	-12.22	-11.82	-12.02

Table S4 Unimolecular rate constants (Log(k)) calculated with the instanton theory and with the CVT/SCT (ISPE correction included).

T (K)	1000/T (1/K)	Instanton	CVT/SCT
140	7.14	7.47	7.01
138	7.25	7.34	6.98
135	7.41	7.23	6.95
125	8.00	6.97	6.80
120	8.33	6.87	6.72
110	9.09	6.71	6.56
100	10.00	6.60	6.41
95	10.53	6.55	6.33
90	11.11	6.51	6.26
85	11.76	6.49	6.20
80	12.50	6.47	6.13
75	13.33	6.45	6.08
70	14.29	6.44	6.02
65	15.38	6.43	5.97
60	16.67	6.44	5.92
55	18.18	6.41	5.88
50	20.00	6.47	5.83
